# Supplementary material for: DMRforPairs: identifying Differentially Methylated Regions between unique samples using array based methylation profiles
Source: BMC Bioinformatics. 2014 May 15;15:141. doi: 10.1186/1471-2105-15-141 (PMC4046028; doi:10.1186/1471-2105-15-141)
Supplement: Additional file 2 — DMRforPairs output for the comparison of A431-MCF7 and NA17018-NA17105. Please start from the HTML files in each folder. Available via the BMC Bioinformatics website. [file 1471-2105-15-141-S2.zip › 1394847754114233_MOESM2_ESM/NA17105M_NA17018F/figures/329.pdf]

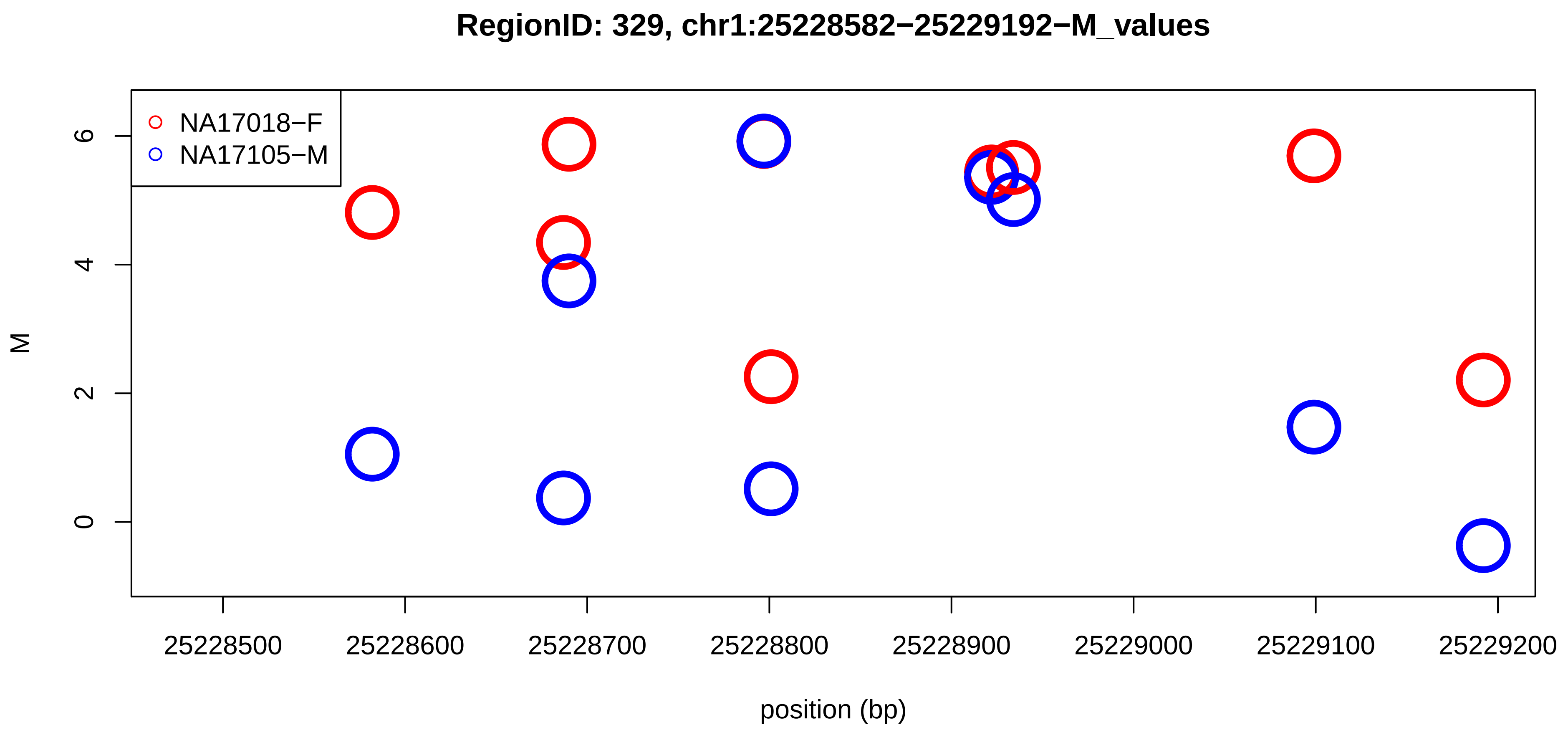

RegionID: 329, chr1:25228582−25229192−M_values
NA17018−F
NA17105−M
M
0
2
4
6
25228500
25228600
25228700
25228800
25228900
25229000
25229100
25229200
position (bp)

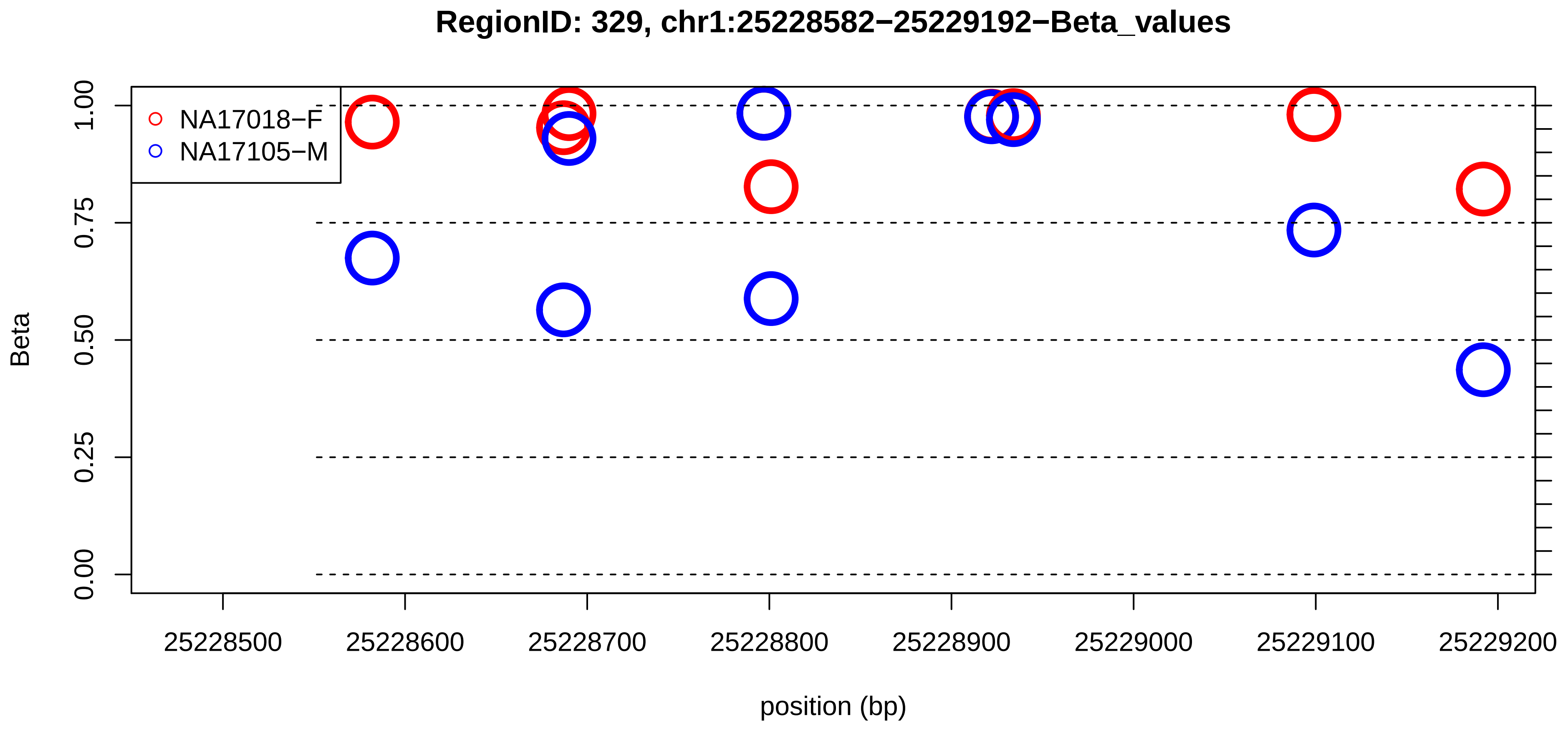

RegionID: 329, chr1:25228582−25229192−Beta_values
NA17018−F
NA17105−M
Beta
0.00
0.25
0.50
0.75
1.00
25228500
25228600
25228700
25228800
25228900
25229000
25229100
25229200
position (bp)
